# Enhancing daily oral PrEP adherence with digital communications: Protocol for a systematic review and meta-analysis

**DOI:** 10.1371/journal.pone.0313322

**Published:** 2024-11-12

**Authors:** Julien Brisson, Mariangela Castro-Arteaga, Dorothy Apedaile, Amaya Perez-Brumer

**Affiliations:** 1 Division of Social and Behavioural Health Sciences, Dalla Lana School of Public Health, University of Toronto, Toronto, Canada; 2 Division of Epidemiology, Dalla Lana School of Public Health, University of Toronto, Toronto, Canada; RAND Corporation, UNITED STATES OF AMERICA

## Abstract

**Introduction:**

Pre-exposure prophylaxis (PrEP) stands as an effective tool in preventing HIV transmission among individuals at risk of HIV infection. However, the effectiveness of daily oral PrEP is contingent on the adherence of its users, which can pose a challenge for many individuals. Various studies have explored different interventions aimed at bolstering PrEP adherence. One recurring type of intervention revolves around digital communication (e.g., SMS, mobile applications) to send reminders for PrEP usage. The objective of our systematic review and meta-analysis is to address the following research question: What is the effectiveness of digital communication interventions in enhancing daily oral PrEP adherence among individuals at a heightened risk of HIV infection? This paper presents our study protocol.

**Method and analysis:**

We will conduct searches across four health-related databases: Embase, PubMed, Web of Science, and PsycINFO. We will also explore other sources, including clinical trials registries and grey literature. Our search will be restricted to original randomized controlled trials published in English, French, and Spanish conducted since 2012, when PrEP was approved, to today. To ensure rigor, three reviewers will perform the systematic review and meta-analysis. This systematic review will adhere to the guidelines outlined in the 2020 Preferred Reporting Items for Systematic Reviews and Meta-Analyses (PRISMA). Our primary outcome of interest is proper daily oral PrEP adherence, which we will measure using association metrics (e.g., odds ratios).

**Discussion:**

This review will offer insights into the effectiveness of utilizing digital communication methods to assist individuals at risk of HIV in improving their PrEP adherence.

**Protocol registration number:**

International Prospective Register for Systematic Reviews (PROSPERO) number CRD42023471269.

## Introduction

The global HIV epidemic remains a substantial public health challenge, marked by an annual incidence exceeding 1.5 million new infections [1, 2]. Of particular concern is the heightened vulnerability of specific key populations to HIV transmission, such as sex workers, people who inject drugs, and gay, bisexual, and other men who have sex with men (GBM) [3]. Among these key populations, adolescents and young adults stand out as a demographic cohort characterized by a disproportionate susceptibility to HIV infection [4, 5]. Notably, the prevalence of new HIV infections is particularly high among adolescent girls and young women in Africa, accounting for an alarming 25% of the total new HIV infections worldwide [6]. In parallel, in the United States, Black and Latine/o GBM face a significantly higher risk of a lifetime of HIV infection compared to other demographic groups [7, 8]. Worldwide, transgender women are also at high risk for HIV acquisition compared to the general population [9]. Sex workers are additionally a key population at increased vulnerability for HIV, particularly in low- and middle-income countries where reported prevalence is reported at 11.8% [10].

In the realm of HIV prevention strategies, pre-exposure prophylaxis (PrEP) has emerged as a pivotal and effective intervention. PrEP involves the use of antiretroviral drugs by individuals who are not living with HIV but may face heightened exposure [11–14]. For example, a systematic review and meta-analysis of 11 trials revealed that PrEP significantly reduced the risk of HIV infection compared to taking a placebo (or no PrEP) over a period ranging from 4 months to 4 years, with a relative risk of 0.46 (95% CI: 0.33–0.66) [15]. However, the effectiveness of oral PrEP hinges significantly on the strict adherence of its users, a challenge that presents difficulties for many individuals [14, 16–18]. For instance, the Partners trial demonstrated that achieving a high adherence rate to PrEP (>80%) was linked to 100% efficacy in preventing HIV transmission (95% CI: 83.7 to 100) [19].

Specific to daily oral formulations of PrEP, the literature details the obstacles faced by individuals in both accessing and adhering to daily oral PrEP regimens [20–24]. These challenges, stemming from a multitude of complex factors [25], including but not limited to stigma [26], cost [27], work schedules [28], physicians’ unwillingness to prescribe PrEP [29, 30], and barriers to PrEP access and other HIV-related services (e.g., HIV testing) [31, 32]. These issues underscore the imperative need for tailored interventions aimed at improving PrEP adherence rates among people who are at increased vulnerability to HIV infection. Especially for people with intersecting social identities known to face increased vulnerability to HIV [3], a need exists to implement tailored strategies that effectively promote and support daily oral PrEP adherence–i.e., taking the prescribed medication as directed. Studies have explored the potential of digital communication methods, such as text messaging and mobile applications, to improve PrEP drug adherence [33, 34]. These studies have produced mixed results in terms of their effectiveness in enhancing daily oral PrEP adherence among users. Therefore, the objective of this systematic review and meta-analysis is to address the following research question: What is the effectiveness of digital communication interventions in enhancing daily oral PrEP adherence among individuals at a heightened risk of HIV infection?

In 2022, Allison et al. [35] conducted a systematic review and meta-analysis that centered on daily PrEP adherence in adolescents and young adults. Simultaneously, in the same year, Zhang et al. [36] conducted a global systematic review and meta-analysis to update and expand upon previous research by quantifying rates of PrEP discontinuation, suboptimal adherence among those who continued PrEP, and re-initiation among those who discontinued PrEP during the observed follow-up period. While these reviews offer comprehensive insights into the prevalence of daily PrEP adherence, they do not address the question of effectiveness in interventions designed to support individuals in adhering to PrEP. Numerous systematic reviews have previously explored interventions related to drug adherence. For instance, in 2014, Marcus et al. [37] conducted a systematic review encompassing various adherence interventions for different medical conditions, including hypertension, latent tuberculosis infection, hyperlipidemia, and contraceptives. Their primary objective was to identify interventions that might potentially enhance PrEP adherence. However, it’s crucial to note that this review was conducted nearly a decade ago and did not specifically analyze interventions involving PrEP.

Studies have explored the use of mobile technologies to improve antiretroviral drug adherence among people living with HIV [38]. Furthermore, there exist separate systematic reviews concerning people living with HIV and their adherence to antiretroviral treatment (ART), including the role of digital communications interventions in increasing adherence to ART [39–44]. Although relevant to the broader context, caution should be exercised when applying the results of these reviews to daily PrEP adherence. This caution is warranted because ART for those with HIV involves a treatment regimen for an existing infection, while PrEP serves as a preventive measure against acquiring an infection.

Our systematic review and meta-analysis will bring forth valuable insights by providing an overview of the current body of literature on the potential effectiveness of digital communication to enhance PrEP adherence to improve strategies for populations at increased vulnerability to HIV.

## Method

### Protocol and registration

This systematic review protocol adheres to the guidelines outlined in the Preferred Reporting Items for Systematic Reviews and Meta-Analyses (PRISMA) for Protocols 2015 (PRISMA-P 2015) (see **S1 File**) [45]. PRISMA-P 2015 offers a comprehensive checklist of essential components designed to facilitate the preparation and development of a systematic review protocol. Following the formulation of our research question, we initiated an initial database search to identify pertinent studies that support the formulation of this protocol. Through this preliminary search, we identified six studies that could potentially answer the research question [33, 46–50].

We have registered this protocol with the International Prospective Register of Systematic Reviews (PROSPERO) under the registration number CRD42023471269. Any necessary adjustments to this protocol will be carried out during the study. In the event of any modifications, they will be promptly reported to PROSPERO and documented in the final version of the article before its publication.

### Search strategy

We will conduct a systematic review by gathering bibliographic data from the following four databases: PubMed, Embase, Web of Science, and PsychInfo. The databases will be searched in October 2023 to identify potential articles for inclusion in the systematic review. To enhance our search strategy, we will also review the reference lists of the included studies and search on clinical trial registry platforms, including WHO International Clinical Trials Registry Platform and ClinicalTrials.gov. Additionally, we will explore grey literature sources and hand search reference lists of the articles included in the review as well as potentially relevant systematic reviews identified during screening. A healthcare librarian has evaluated and provided valuable feedback on how to conduct our search strategy.

### Eligibility criteria

The systematic review question in this study is framed using the PICOS (Population, Intervention, Comparison, Outcome, Study Design) framework, as per the method outlined in the Cochrane Handbook for Systematic Reviews of Interventions version 6.3 [51]. The objective of this systematic review and meta-analysis is to critically assess the effectiveness of digital communication interventions in enhancing PrEP adherence. See **Table 1** for an example of keywords structured under the PICOS framework to be used in the search strategy. The PICOS framework guiding our research is defined as follows:

**Table 1:** Example of keywords to be used in the search strategy organized into blocks.

Blocks (PICOS)	
#1	“HIV” OR “HIV infection” OR “pre-exposure prophylaxis” OR “antiviral agents” OR “antiviral drugs”
P	
#2	“internet” OR “telephone” OR “mobile application” OR “communications media” OR “mobile phone” OR “electronic communication”
I	
#3	“standard of care”
C	
#4	“adherence”
O	
#5	“Randomized controlled trial” OR “clinical trials”
S	
Search string	(#1) AND (#2) AND (#3) AND (#4) AND (#5)
Search refinements	Language: English, French, Spanish Publication year: 2012–2023

**Note 1:** PICOS stands for Population, Intervention, Comparison, Outcome, Study Design. It is a framework created to define the issue addressed by the systematic review.

**Note 2**: Each database has its unique research logistics. The keywords in the table are general and not tailored to any specific database. The keywords are provided for informational purposes to demonstrate how we will conduct the systematic review using the PICOS framework and will be adapted as needed.

### Population

Our population of interest comprises individuals at a high risk of HIV infection through sexual relations, including cisgender women, GBM, transgender and gender non-binary people, sex workers, and adolescents and young individuals, as PrEP is intended for the prevention of HIV transmission.

### Intervention

Within the realm of digital communications, eligible interventions encompass various modalities such as mobile applications, text messaging, phone calls, and email communications.

### Comparison group

The comparison group represents the standard of care, denoting cases where no digital communication intervention is applied.

### Outcome

Our primary outcome of interest pertains to daily oral PrEP adherence, a critical factor in evaluating the effectiveness of digital communication interventions in this context.

### Study design

To determine intervention efficacy with the highest level of rigor, we prioritize randomized clinical trials (RCTs) as the preferred study design. These RCTs compare the effects of digital communication interventions against the standard of care (i.e., no intervention), providing a robust foundation for assessing the impact of the intervention on PrEP adherence. The RCT design was selected due to the focus of this review on the effectiveness of a specific intervention compared to standard of care. While observational study designs can offer valuable insights, they rely on non-randomized exposure assignment, influenced by patient preferences, healthcare provider decisions, and other external factors. This non-random allocation introduces the potential for bias and confounding, undermining internal validity. By including only RCTs in our systematic review and meta-analysis, we aimed to enhance the overall quality and robustness of the synthesized evidence base.

Inclusion criteria for studies are as follows:

1. Study Design: Eligible studies will be limited to RCTs with a comparator group, a prerequisite for assessing the efficacy of the digital communication intervention.
2. PrEP Modality: Included studies will focus exclusively on daily oral PrEP to facilitate meaningful comparisons. Studies involving on-demand/event driven oral PrEP use, vaginal ring PrEP or long-acting injectable PrEP, which exhibit distinct adherence dynamics, will not be considered.
3. Geographical and Demographic Considerations: Given the relatively limited volume of research on digital communication interventions for PrEP, this review will not impose geographical restrictions. It will encompass diverse demographic groups at elevated risk of HIV transmission, including, but not limited to, women, GBM, and young individuals.
4. Outcome of Interest: The outcome of interest must relate specifically to daily oral PrEP adherence. There are different PrEP adherence measurement methods, such as electronic bottle pill counts and blood or urine samples (biological samples being the most robust form of measurement of PrEP adherence). All forms of PrEP adherence measurement will be deemed eligible for inclusion.
5. Publication Source: Eligible studies must be published in peer-reviewed journals or databases (e.g., ClinicalTrials.gov) to ensure research quality and rigor.
6. Language: Included studies should be available in English, French, or Spanish, languages that can be comprehensively evaluated by the research team.
7. Publication Timeframe: Given that PrEP was initially approved by the US FDA in 2012, this systematic review will encompass studies published between 2012 and 2023.

Regarding exclusion criteria, we will exclude the following studies from our research:

1. Exclusion of Non-Digital Communication Interventions: Studies on PrEP adherence that focus on interventions other than digital communications, such as monetary incentives, will be excluded.
2. Duplicate Data: Exclude studies that present the same dataset or results as another included study to avoid redundancy.
3. Non-Original Research: Exclude studies that are not original research, such as review articles, editorials, commentaries, or letters to the editor.
4. Studies with Ethical Concerns: Exclude studies with substantial ethical concerns, such as studies with serious violations of research ethics or that did not obtain institutional research ethics approval.

### Review process

1. Reviewer training: Researchers responsible for evaluating article eligibility will receive training in the application of predefined inclusion and exclusion criteria. This training will involve in-depth comprehension of the criteria and guidelines, along with hands-on exercises to ensure uniform and precise article selection during the review process. Furthermore, a pilot practice involving 50 references will be conducted as part of the training. The goal is to enhance inter-rater reliability in the review [52].
2. Screening: The literature obtained will be exported in the online software Covidence [53] where duplicate records will be identified and removed. The screening process will comprise two stages: initial screening of titles and abstracts, followed by a subsequent full-text screening of selected articles. In the initial stage, one reviewer will assess the titles and abstracts for compliance with the predefined inclusion criteria. Subsequently, a second reviewer will independently review the same titles and abstracts to validate the initial reviewer’s assessments. In cases where discrepancies arise, a consensus will be reached through consultation with a third reviewer. A similar approach will be implemented for the full-text screening, with one reviewer initially examining the full texts, followed by independent validation by a second reviewer to determine their eligibility. Any disparities in assessments during the full-text screening will also be resolved through consensus involving the third reviewer.
3. Data extraction: Once the full texts have been identified, all three reviewers will use Cochrane’s Data Extraction and Assessment Form template for RCTs (see **S2 File**) to assess each study selected for inclusion in the review. Following this, one reviewer will compare the data extraction forms for each study to ensure consensus in the data extraction process. In cases where discrepancies emerge, the three reviewers will come together to reach a consensus.

### Data analysis

The data analysis will be conducted in the statistical software R. Our primary objective is to assess the effectiveness of digital communication interventions on daily oral PrEP adherence. To achieve this, we will conduct a comprehensive comparison between the intervention and control groups across the included studies. Specifically, we will calculate the measure of association (relative risk) by pooling data from all the studies in our meta-analysis. This analysis will allow us to quantitatively evaluate the impact of these interventions on oral PrEP adherence.

To provide a clear and precise estimate of the intervention’s effect, we will present efficacy values along with 95% confidence intervals. These confidence intervals will indicate the range within which we can be reasonably certain the true effect lies. Such presentation is essential for understanding the statistical significance and clinical relevance of the intervention’s impact on oral PrEP adherence. To assess the magnitude of heterogeneity between the included studies, we will measure an index of heterogeneity (I^2^) to indicate the percentage of the total variation across studies.

**Fig 1:**
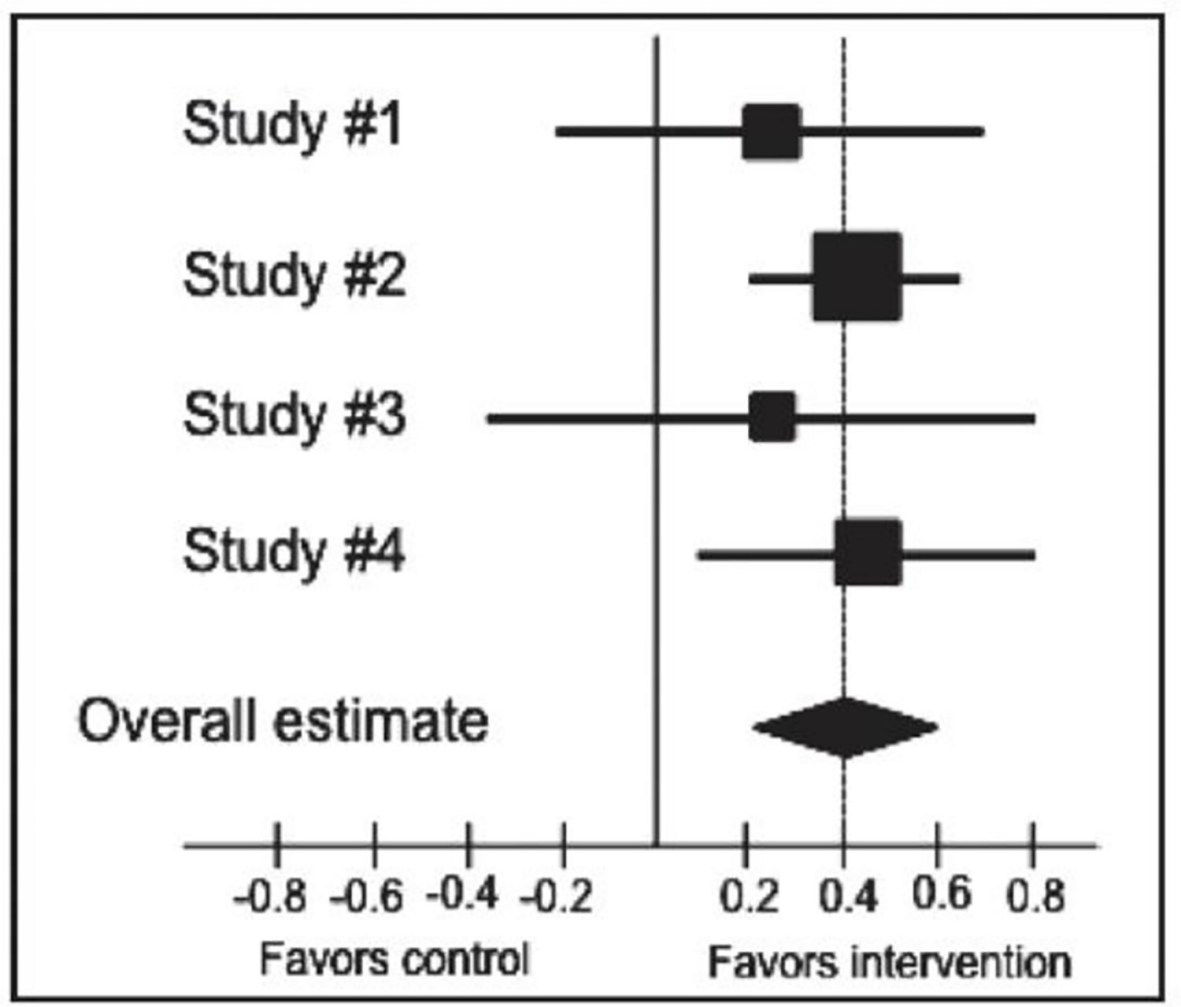
Example of a forest plot for meta-analysis [54].

To enhance the interpretability of our findings, we will create a forest plot (**Fig 1**). This graphical representation will offer a visual summary of the effect sizes and their precision across the included studies. It will provide a comprehensive overview of the intervention’s impact and highlight any variations or trends in the data.

Additionally, to explore potential variations in the intervention’s effectiveness across different contexts and populations, we will conduct subgroup analyses using a mixed effects model. While the ability to conduct subgroup analyses is dependent on the number of articles identified in this systematic review, we plan to conduct sub-group analyses along the following variables: HIV risk key population (e.g., youth, transgender women, GBM), age, sex/gender, type of digital communication (e.g., text messaging, mobile apps), and geographical location. By considering these factors, we aim to identify any subgroup-specific effects and gain insights into the broader applicability of the interventions.

### Assessment of quality and risk of bias

We will assess the risk of bias in each study using the Cochrane Collaboration Risk of Bias Tool (RoB 2.0) [55]. This tool is designed to comprehensively evaluate bias in six key aspects: randomization, deviations from intended interventions, missing outcomes, outcome measurement, selective reporting of results, and the timing of participant identification and recruitment.

### Ethics aspects and plans for dissemination

Given the study’s design characteristics, ethical committee approval was not required. The findings from this systematic review will be shared through peer-reviewed publications, as well as through various platforms including symposia and conferences within the relevant field.

## Discussion

From 2012, the year that PrEP first became approved in the US, to 2017, there was a 56% annual increase in PrEP use [56]. While countries have slowly been making PrEP more accessible, there are still significant access barriers to PrEP globally [57, 58]. Despite novel PrEP technologies such as vaginal ring PrEP [59] and long-acting injectable cabotegravir [60] slowly becoming more available, oral PrEP may be preferred by some individuals. Consequently, identifying effective measures to support individuals who may encounter challenges with oral PrEP adherence is paramount. Research has explored various interventions aimed at improving adherence, including monetary incentives [61], counseling [62], and HIV self-testing [63]. These studies demonstrate varying success rates, emphasizing the ongoing need to explore diverse strategies for personalized support for PrEP users.

It is essential to acknowledge the inherent limitations of this systematic review. Notably, the data synthesized for this analysis are drawn from studies conducted across diverse populations, each characterized by unique demographic attributes such as age, gender, sexuality, and geographical location. While this diversity can offer valuable insights into PrEP adherence across different contexts, it introduces complexities when attempting to draw overarching conclusions. The variations in demographic characteristics among these populations may obscure nuanced differences in the effectiveness of digital communication strategies for promoting PrEP adherence among specific subgroups, making it challenging to provide tailor-made recommendations for each demographic. Moreover, the presence of these demographic disparities could potentially impact the applicability of our findings to broader populations or specific at-risk groups.

Our objective is to disseminate the results of this systematic review and meta-analysis through publication in a public health journal. We will present the results following the guidelines outlined in the 2020 Preferred Reporting Items for Systematic Reviews and Meta-Analyses (PRISMA). By doing so, we aim to furnish valuable insights into the effectiveness of digital communication strategies in bolstering oral adherence to PrEP. This information possesses the potential to inform and guide public health practitioners in their endeavors to optimize PrEP utilization, with the goal of reducing the incidence of HIV infection.

## Supporting information

S1 File Checklist. Preferred Reporting Items for Systematic Reviews and Meta-Analyses (PRISMA) checklist.
(PDF)

S2 File Cochrane’s data extraction and assessment form template for RCTs. (DOC)

## References

[1] Mahy M, Marsh K, Sabin K, Wanyeki I, Daher J, Ghys PD. HIV estimates through 2018: data for decision-making. AIDS. 2019 Dec 15;33(Supplement 3):S203–11. doi: 10.1097/QAD.0000000000002321 31343430 PMC6919227

[2] Frank TD, Carter A, Jahagirdar D, Biehl MH, Douwes-Schultz D, Larson SL, et al. Global, regional, and national incidence, prevalence, and mortality of HIV, 1980–2017, and forecasts to 2030, for 195 countries and territories: a systematic analysis for the Global Burden of Diseases, Injuries, and Risk Factors Study 2017. Lancet HIV. 2019 Dec;6(12):e831–59. doi: 10.1016/S2352-3018(19)30196-1 31439534 PMC6934077

[3] World Health Organization. Consolidated guidelines on HIV prevention, diagnosis, treatment and care for key populations [Internet]. 2016 update. Geneva: World Health Organization; 2016 [cited 2024 Mar 25]. 155 p. Available from: https://iris.who.int/handle/10665/246200

[4] Pettifor A, Stoner M, Pike C, Bekker LG. Adolescent lives matter: preventing HIV in adolescents. Curr Opin HIV AIDS. 2018 May;13(3):265–73. doi: 10.1097/COH.0000000000000453 29528850 PMC5902132

[5] Bekker LG, Hosek S. HIV and adolescents: focus on young key populations. J Int AIDS Soc. 2015 Feb;18:20076.

[6] Celum CL, Delany‐Moretlwe S, Baeten JM, Van Der Straten A, Hosek S, Bukusi EA, et al. HIV pre‐exposure prophylaxis for adolescent girls and young women in Africa: from efficacy trials to delivery. J Int AIDS Soc. 2019 Jul;22(S4):e25298. 31328444 10.1002/jia2.25298 PMC6643076

[7] Singh S, Song R, Johnson AS, McCray E, Hall HI. HIV Incidence, Prevalence, and Undiagnosed Infections in U.S. Men Who Have Sex With Men. Ann Intern Med. 2018 May 15;168(10):685. doi: 10.7326/M17-2082 29554663

[8] Hess KL, Hu X, Lansky A, Mermin J, Hall HI. Lifetime risk of a diagnosis of HIV infection in the United States. Ann Epidemiol. 2017 Apr;27(4):238–43. doi: 10.1016/j.annepidem.2017.02.003 28325538 PMC5524204

[9] Stutterheim SE, Van Dijk M, Wang H, Jonas KJ. The worldwide burden of HIV in transgender individuals: An updated systematic review and meta-analysis. Lima VD, editor. PLOS ONE. 2021 Dec 1;16(12):e0260063. doi: 10.1371/journal.pone.0260063 34851961 PMC8635361

[10] Baral S, Beyrer C, Muessig K, Poteat T, Wirtz AL, Decker MR, et al. Burden of HIV among female sex workers in low-income and middle-income countries: a systematic review and meta-analysis. Lancet Infect Dis. 2012 Jul;12(7):538–49. doi: 10.1016/S1473-3099(12)70066-X 22424777

[11] Grant RM, Lama JR, Anderson PL, McMahan V, Liu AY, Vargas L, et al. Preexposure Chemoprophylaxis for HIV Prevention in Men Who Have Sex with Men. N Engl J Med. 2010 Dec 30;363(27):2587–99. doi: 10.1056/NEJMoa1011205 21091279 PMC3079639

[12] McCormack S, Dunn DT, Desai M, Dolling DI, Gafos M, Gilson R, et al. Pre-exposure prophylaxis to prevent the acquisition of HIV-1 infection (PROUD): effectiveness results from the pilot phase of a pragmatic open-label randomised trial. The Lancet. 2016 Jan;387(10013):53–60. doi: 10.1016/S0140-6736(15)00056-2 26364263 PMC4700047

[13] Thigpen MC, Kebaabetswe PM, Paxton LA, Smith DK, Rose CE, Segolodi TM, et al. Antiretroviral Preexposure Prophylaxis for Heterosexual HIV Transmission in Botswana. N Engl J Med. 2012 Aug 2;367(5):423–34. doi: 10.1056/NEJMoa1110711 22784038

[14] Dimitrov DT, Mâsse BR, Donnell D. PrEP Adherence Patterns Strongly Affect Individual HIV Risk and Observed Efficacy in Randomized Clinical Trials. JAIDS J Acquir Immune Defic Syndr. 2016 Aug 1;72(4):444–51. doi: 10.1097/QAI.0000000000000993 26990823 PMC4925182

[15] Chou R, Evans C, Hoverman A, Sun C, Dana T, Bougatsos C, et al. Preexposure Prophylaxis for the Prevention of HIV Infection: Evidence Report and Systematic Review for the US Preventive Services Task Force. JAMA. 2019 Jun 11;321(22):2214. doi: 10.1001/jama.2019.2591 31184746

[16] Grant RM, Anderson PL, McMahan V, Liu A, Amico KR, Mehrotra M, et al. Uptake of pre-exposure prophylaxis, sexual practices, and HIV incidence in men and transgender women who have sex with men: a cohort study. Lancet Infect Dis. 2014 Sep;14(9):820–9. doi: 10.1016/S1473-3099(14)70847-3 25065857 PMC6107918

[17] Fonner VA, Dalglish SL, Kennedy CE, Baggaley R, O’Reilly KR, Koechlin FM, et al. Effectiveness and safety of oral HIV preexposure prophylaxis for all populations. AIDS. 2016 Jul 31;30(12):1973–83. doi: 10.1097/QAD.0000000000001145 27149090 PMC4949005

[18] Sidebottom D, Ekström AM, Strömdahl S. A systematic review of adherence to oral pre-exposure prophylaxis for HIV–how can we improve uptake and adherence? BMC Infect Dis. 2018 Dec;18(1):581. doi: 10.1186/s12879-018-3463-4 30445925 PMC6240194

[19] Haberer JE, Baeten JM, Campbell J, Wangisi J, Katabira E, Ronald A, et al. Adherence to Antiretroviral Prophylaxis for HIV Prevention: A Substudy Cohort within a Clinical Trial of Serodiscordant Couples in East Africa. Siegfried Neditor. PLoS Med. 2013 Sep 10;10(9):e1001511. doi: 10.1371/journal.pmed.1001511 24058300 PMC3769210

[20] Hosek S, Henry-Reid L. PrEP and Adolescents: The Role of Providers in Ending the AIDS Epidemic. Pediatrics. 2020 Jan 1;145(1):e20191743. doi: 10.1542/peds.2019-1743 31857381

[21] Griffin S. HIV: Two in three people have trouble getting PrEP, finds survey. BMJ. 2022 Nov 4;o2667. doi: 10.1136/bmj.o2667 36332912

[22] Goparaju L, Praschan NC, Jeanpiere LW, Experton LS, Young MA, Kassaye S. Stigma, Partners, Providers and Costs: Potential Barriers to PrEP Uptake among US Women. J AIDS Clin Res [Internet]. 2017 [cited 2024 Mar 7];08(09). Available from: https://www.omicsonline.org/open-access/stigma-partners-providers-and-costs-potential-barriers-to-prep-uptakeamong-us-women-2155-6113-1000730.php?aid=93928 doi: 10.4172/2155-6113.1000730 29201531 PMC5708581

[23] Edeza A, Karina Santamaria E, Valente PK, Gomez A, Ogunbajo A, Biello K. Experienced barriers to adherence to pre-exposure prophylaxis for HIV prevention among MSM: a systematic review and meta-ethnography of qualitative studies. AIDS Care. 2021 Jun 3;33(6):697–705. doi: 10.1080/09540121.2020.1778628 32530302

[24] Hubach RD, Currin JM, Sanders CA, Durham AR, Kavanaugh KE, Wheeler DL, et al. Barriers to Access and Adoption of Pre-Exposure Prophylaxis for the Prevention of HIV Among Men Who Have Sex With Men (MSM) in a Relatively Rural State. AIDS Educ Prev. 2017 Aug;29(4):315–29. doi: 10.1521/aeap.2017.29.4.315 28825858

[25] Wood S, Gross R, Shea JA, Bauermeister JA, Franklin J, Petsis D, et al. Barriers and Facilitators of PrEP Adherence for Young Men and Transgender Women of Color. AIDS Behav. 2019 Oct;23(10):2719–29. doi: 10.1007/s10461-019-02502-y 30993479 PMC6790163

[26] Calabrese SK. Understanding, Contextualizing, and Addressing PrEP Stigma to Enhance PrEP Implementation. Curr HIV/AIDS Rep. 2020 Dec;17(6):579–88. doi: 10.1007/s11904-020-00533-y 32965576

[27] Kay ES, Pinto RM. Is Insurance a Barrier to HIV Preexposure Prophylaxis? Clarifying the Issue. Am J Public Health. 2020 Jan;110(1):61–4. doi: 10.2105/AJPH.2019.305389 31725314 PMC6893325

[28] Van Der Elst EM, Mbogua J, Operario D, Mutua G, Kuo C, Mugo P, et al. High Acceptability of HIV Pre-exposure Prophylaxis but Challenges in Adherence and Use: Qualitative Insights from a Phase I Trial of Intermittent and Daily PrEP in At-Risk Populations in Kenya. AIDS Behav. 2013 Jul;17(6):2162–72. doi: 10.1007/s10461-012-0317-8 23080358 PMC3690654

[29] Turner L, Roepke A, Wardell E, Teitelman AM. Do You PrEP? A Review of Primary Care Provider Knowledge of PrEP and Attitudes on Prescribing PrEP. J Assoc Nurses AIDS Care. 2018 Jan;29(1):83–92. doi: 10.1016/j.jana.2017.11.002 29274655 PMC7653672

[30] Vega-Ramirez H, Torres TS, Guillen-Diaz C, Pimenta C, Diaz-Sosa D, Konda KA, et al. Awareness, knowledge, and attitudes related to HIV pre-exposure prophylaxis and other prevention strategies among physicians from Brazil and Mexico: cross-sectional web-based survey. BMC Health Serv Res. 2022 Dec;22(1):532. doi: 10.1186/s12913-022-07900-y 35459177 PMC9027096

[31] Irungu EM, Baeten JM. PrEP rollout in Africa: status and opportunity. Nat Med. 2020 May;26(5):655–64. doi: 10.1038/s41591-020-0872-x 32405065

[32] Bavinton BR, Grulich AE. HIV pre-exposure prophylaxis: scaling up for impact now and in the future. Lancet Public Health. 2021 Jul;6(7):e528–33. doi: 10.1016/S2468-2667(21)00112-2 34087117

[33] Haberer JE, Bukusi EA, Mugo NR, Pyra M, Kiptinness C, Oware K, et al. Effect of SMS reminders on PrEP adherence in young Kenyan women (MPYA study): a randomised controlled trial. Lancet HIV. 2021 Mar;8(3):e130–7. doi: 10.1016/S2352-3018(20)30307-6 33662265 PMC8289198

[34] Sharpe JD, Kamara MT. A systematic evaluation of mobile apps to improve the uptake of and adherence to HIV pre-exposure prophylaxis. Sex Health. 2018;15(6):587. doi: 10.1071/SH18120 30347177

[35] Allison BA, Widman L, Stewart JL, Evans R, Perry M. Adherence to Pre-Exposure Prophylaxis in Adolescents and Young Adults: A Systematic Review and Meta-Analysis. J Adolesc Health. 2022 Jan;70(1):28–41. doi: 10.1016/j.jadohealth.2021.04.001 34059426

[36] Zhang J, Li C, Xu J, Hu Z, Rutstein SE, Tucker JD, et al. Discontinuation, suboptimal adherence, and reinitiation of oral HIV pre-exposure prophylaxis: a global systematic review and meta-analysis. Lancet HIV. 2022 Apr;9(4):e254–68. doi: 10.1016/S2352-3018(22)00030-3 35364026 PMC9124596

[37] Marcus J, Buisker T, Horvath T, Amico K, Fuchs J, Buchbinder S, et al. Helping our patients take HIV pre‐exposure prophylaxis (PrEP): a systematic review of adherence interventions. HIV Med. 2014 Aug;15(7):385–95. doi: 10.1111/hiv.12132 24580813 PMC4107052

[38] DeFulio A, Devoto A, Traxler H, Cosottile D, Fingerhood M, Nuzzo P, et al. Smartphone-based incentives for promoting adherence to antiretroviral therapy: A randomized controlled trial. Prev Med Rep. 2021 Mar;21:101318. doi: 10.1016/j.pmedr.2021.101318 33511028 PMC7815813

[39] Casale M, Carlqvist A, Cluver L. Recent Interventions to Improve Retention in HIV Care and Adherence to Antiretroviral Treatment Among Adolescents and Youth: A Systematic Review. AIDS Patient Care STDs. 2019 Jun;33(6):237–52. doi: 10.1089/apc.2018.0320 31166783 PMC6588099

[40] Hudelson C, Cluver L. Factors associated with adherence to antiretroviral therapy among adolescents living with HIV/AIDS in low- and middle-income countries: a systematic review. AIDS Care. 2015 Jul 3;27(7):805–16. doi: 10.1080/09540121.2015.1011073 25702789

[41] Kim SH, Gerver SM, Fidler S, Ward H. Adherence to antiretroviral therapy in adolescents living with HIV: systematic review and meta-analysis. AIDS. 2014 Aug 24;28(13):1945–56. doi: 10.1097/QAD.0000000000000316 24845154 PMC4162330

[42] Laurenzi CA, Du Toit S, Ameyan W, Melendez‐Torres G, Kara T, Brand A, et al. Psychosocial interventions for improving engagement in care and health and behavioural outcomes for adolescents and young people living with HIV: a systematic review and meta‐analysis. J Int AIDS Soc. 2021 Aug;24(8):e25741. doi: 10.1002/jia2.25741 34338417 PMC8327356

[43] Reif LK, Abrams EJ, Arpadi S, Elul B, McNairy ML, Fitzgerald DW, et al. Interventions to Improve Antiretroviral Therapy Adherence Among Adolescents and Youth in Low- and Middle-Income Countries: A Systematic Review 2015–2019. AIDS Behav. 2020 Oct;24(10):2797–810. doi: 10.1007/s10461-020-02822-4 32152815 PMC7223708

[44] Ridgeway K, Dulli LS, Murray KR, Silverstein H, Dal Santo L, Olsen P, et al. Interventions to improve antiretroviral therapy adherence among adolescents in low- and middle-income countries: A systematic review of the literature. Paraskevis D, editor. PLOS ONE. 2018 Jan 2;13(1):e0189770. doi: 10.1371/journal.pone.0189770 29293523 PMC5749726

[45] Shamseer L, Moher D, Clarke M, Ghersi D, Liberati A, Petticrew M, et al. Preferred reporting items for systematic review and meta-analysis protocols (PRISMA-P) 2015: elaboration and explanation. BMJ. 2015 Jan 2;349(jan02 1):g7647–g7647. doi: 10.1136/bmj.g7647 25555855

[46] Liu AY, Vittinghoff E, Von Felten P, Rivet Amico K, Anderson PL, Lester R, et al. Randomized Controlled Trial of a Mobile Health Intervention to Promote Retention and Adherence to Preexposure Prophylaxis Among Young People at Risk for Human Immunodeficiency Virus: The EPIC Study. Clin Infect Dis. 2019 May 30;68(12):2010–7. doi: 10.1093/cid/ciy810 30239620 PMC6541706

[47] Moore DJ, Jain S, Dubé MP, Daar ES, Sun X, Young J, et al. Randomized Controlled Trial of Daily Text Messages to Support Adherence to Preexposure Prophylaxis in Individuals at Risk for Human Immunodeficiency Virus: The TAPIR Study. Clin Infect Dis. 2018 May 2;66(10):1566–72. doi: 10.1093/cid/cix1055 29228144 PMC6248545

[48] Songtaweesin WN, Kawichai S, Phanuphak N, Cressey TR, Wongharn P, Saisaengjan C, et al. Youth‐friendly services and a mobile phone application to promote adherence to pre‐exposure prophylaxis among adolescent men who have sex with men and transgender women at‐risk for HIV in Thailand: a randomized control trial. J Int AIDS Soc. 2020 Sep;23(S5):e25564. doi: 10.1002/jia2.25564 32869511 PMC7459171

[49] Van Den Elshout MAM, Hoornenborg E, Achterbergh RCA, Coyer L, Anderson PL, Davidovich U, et al. Improving adherence to daily preexposure prophylaxis among MSM in Amsterdam by providing feedback via a mobile application. AIDS. 2021 Sep 1;35(11):1823–34. doi: 10.1097/QAD.0000000000002949 34001705

[50] Whiteley L, Craker L, Haubrick KK, Arnold T, Mena L, Olsen E, et al. The Impact of a Mobile Gaming Intervention to Increase Adherence to Pre-exposure Prophylaxis. AIDS Behav. 2021 Jun;25(6):1884–9. doi: 10.1007/s10461-020-03118-3 33483897 PMC8085097

[51] Higgins JP. Cochrane Handbook for Systematic Reviews of Interventions version 6.3. London: Cochrane; 2022.

[52] Belur J, Tompson L, Thornton A, Simon M. Interrater Reliability in Systematic Review Methodology: Exploring Variation in Coder Decision-Making. Sociol Methods Res. 2021 May;50(2):837–65.

[53] Veritas Health Innovation. Covidence systematic review software [Internet]. Melbourne, Australia; Available from: www.covidence.org

[54] Impellizzeri FM, Bizzini M. Systematic review and meta-analysis: a primer. Int J Sports Phys Ther. 2012 Oct;7(5):493–503. 23091781 PMC3474302

[55] Savović J, Weeks L, Sterne JA, Turner L, Altman DG, Moher D, et al. Evaluation of the Cochrane Collaboration’s tool for assessing the risk of bias in randomized trials: focus groups, online survey, proposed recommendations and their implementation. Syst Rev. 2014 Dec;3(1):37. doi: 10.1186/2046-4053-3-37 24731537 PMC4022341

[56] Sullivan PS, Stephenson R, Hirshfield S, Mehta CC, Zahn R, Bauermeister JA, et al. Behavioral Efficacy of a Sexual Health Mobile App for Men Who Have Sex With Men: Randomized Controlled Trial of Mobile Messaging for Men. J Med Internet Res. 2022 Feb 2;24(2):e34574. doi: 10.2196/34574 35025755 PMC8851328

[57] Kebede S, Brazier E, Freeman AM, Muwonge TR, Choi JY, De Waal R, et al. PrEP Availability Among Health Facilities Participating in the Global IeDEA Consortium. AIDS [Internet]. 2023 Dec 22 [cited 2024 Mar 9]; Available from: https://journals.lww.com/10.1097/QAD.0000000000003824 10.1097/QAD.0000000000003824 PMC10939841 38133656

[58] Moyo PL, Nunu WN. Oral Pre-exposure Prophylaxis Uptake and Acceptability Among Men Who Have Sex With Men: A Scoping Review of the Literature. Am J Mens Health. 2023 Sep;17(5):15579883231201729. doi: 10.1177/15579883231201729 37776162 PMC10541771

[59] Reidy M, Gardiner E, Pretorius C, Glaubius R, Torjesen K, Kripke K. Evaluating the potential impact and cost-effectiveness of dapivirine vaginal ring pre-exposure prophylaxis for HIV prevention. Mugo NR, editor. PLOS ONE. 2019 Jun 26;14(6):e0218710. doi: 10.1371/journal.pone.0218710 31242240 PMC6594614

[60] Durham SH, Milam A, Waer D, Chahine EB. Cabotegravir: The First Long-Acting Injectable for HIV Preexposure Prophylaxis. Ann Pharmacother. 2023 Mar;57(3):306–16. doi: 10.1177/10600280221102532 35778802

[61] Celum CL, Gill K, Morton JF, Stein G, Myers L, Thomas KK, et al. Incentives conditioned on tenofovir levels to support PrEP adherence among young South African women: a randomized trial. J Int AIDS Soc. 2020 Nov;23(11):e25636. doi: 10.1002/jia2.25636 33247553 PMC7695999

[62] Mayer KH, Safren SA, Elsesser SA, Psaros C, Tinsley JP, Marzinke M, et al. Optimizing Pre-Exposure Antiretroviral Prophylaxis Adherence in Men Who Have Sex with Men: Results of a Pilot Randomized Controlled Trial of “Life-Steps for PrEP.” AIDS Behav. 2017 May;21(5):1350–60. 27848089 10.1007/s10461-016-1606-4 PMC5380582

[63] Mujugira A, Nakyanzi A, Nabaggala MS, Muwonge TR, Ssebuliba T, Bagaya M, et al. Effect of HIV Self-Testing on PrEP Adherence Among Gender-Diverse Sex Workers in Uganda: A Randomized Trial. JAIDS J Acquir Immune Defic Syndr. 2022 Apr 1;89(4):381–9. doi: 10.1097/QAI.0000000000002895 34954718 PMC8860206

